# Supplementary material for: Risk and protective factors for post-traumatic stress among New Zealand military personnel: A cross sectional study
Source: PLoS One. 2020 Apr 17;15(4):e0231460. doi: 10.1371/journal.pone.0231460 (PMC7164978; doi:10.1371/journal.pone.0231460)
Supplement: S1 Table — (DOCX) [file pone.0231460.s001.docx]

|  | | | | | | | | |
| --- | --- | --- | --- | --- | --- | --- | --- | --- |
|  | **Type of Event** | **Event ever happened** | | **Among those event happened, perceived threat to life or threat of serious injury** | | **Among those event happened, physical injury** | | **Met DSM-IV criterion** |
|  |  | **N** | **%*** | **N** | **%**** | **N** | **%**** | **%***** |
| **1** | **War Zone** | 1006 | 55.4 | 736 | 73.2 | 27 | 2.7 | 40.5 |
| **2** | **Serious Accident** | 576 | 31.7 | 418 | 72.6 | 175 | 30.4 | 23.0 |
| **3** | **Natural Disaster** | 707 | 38.9 | 311 | 44.0 | 5 | 0.7 | 17.1 |
| **4** | **Life Threatening Illness** | 302 | 16.6 | 232 | 76.8 | 0 | 0.0 | 12.8 |
| **5** | **Childhood Physical Abuse** | 601 | 33.1 | 165 | 27.5 | 36 | 6.0 | 9.1 |
| **6** | **Mugged or Assaulted** | 712 | 39.2 | 339 | 47.6 | 85 | 11.9 | 18.7 |
| **7** | **Childhood Sexual Abuse** | 272 | 15.0 | 35 | 12.9 | 10 | 3.7 | 1.9 |
| **8** | **Other Fearful Situations** | 622 | 34.2 | 0 | 0.0 | 117 | 18.8 | 0.0 |
| **9** | **Death of Family Member in Violent Event** | 378 | 20.8 | 0 | 0.0 | 12 | 3.2 | 0.0 |
| **10** | **Witnessed Traumatic Event** | 870 | 47.9 | 0 | 0.0 | 0 | 0.0 | 0.0 |
