# Supplementary material for: Risk and protective factors for post-traumatic stress among New Zealand military personnel: A cross sectional study
Source: PLoS One. 2020 Apr 17;15(4):e0231460. doi: 10.1371/journal.pone.0231460 (PMC7164978; doi:10.1371/journal.pone.0231460)
Supplement: Attachment — Submitted filename: PONE-D-19-23666_Editor comments.pdf [file pone.0231460.s002.pdf]

PLOS ONE

Post-Traumatic Stress among New Zealand military personnel: a cross sectional study

--Manuscript Draft--

**Manuscript Number:**	PONE-D-19-23666
**Article Type:**	Research Article
**Full Title:**	Post-Traumatic Stress among New Zealand military personnel: a cross sectional study
**Short Title:**	PTSD in New Zealand Veterans
**Corresponding Author:**	David McBride<br>University of Otago<br>Dunedin, Otago NEW ZEALAND
**Keywords:**	Military Personnel; Post-Traumatic Stress (PTS); Psychological Flexibility; Sleep; Trauma
**Abstract:**	Background<br><br>Post-traumatic stress (PTS) is prevalent among military personnel. Knowledge of the protective and harmful factors associated with PTS in this population may assist with identifying personnel who would benefit from increased or targeted support.<br><br>Aims<br><br>To examine factors associated with PTS among New Zealand military personnel.<br><br>Methods<br><br>For this cross-sectional study, currently serving and retired military personnel were invited to complete a questionnaire. The questionnaire included a measure of PTS (the Military Post-traumatic Stress Disorder Checklist; PCL-M), where scores ≥30 indicate the experience of significant PTS symptoms and scores ≥45 indicate a presumptive clinical diagnosis of post-traumatic stress. Potentially protective and harmful factors associated with PTS were examined using logistic regression modelling.<br><br>Results<br><br>1817 military personnel completed the questionnaire. PCL-M scores were ≥30 for 549 (30%) participants and ≥45 for 179 (10%) participants. Exposure to trauma was most strongly associated with PCL-M scores ≥45 (OR 3.34, 95% CI 1.54-7.27, p<0.01). Higher PCL-M scores were also associated with older age, male sex, and Māori ethnicity (New Zealand's indigenous population). Factors associated with lower PCL-M scores were greater length of service, psychological flexibility, and better quality sleep.<br><br>Conclusions<br><br>PTS was found to be prevalent among New Zealand military personnel. The experience of trauma was strongly associated with PTS. However, factors such as psychological flexibility (the ability to adapt to changes in circumstances) and good sleep were protective, suggesting that these factors could be key targets for interventions designed to reduce PTS among military personnel in New Zealand.
**Order of Authors:**	Amy Richardson
	Gagan Gurung
	Ari Samaranayaka
	Dianne Gardner
	Brandon deGraaf
	Emma H. Wyeth
	Sarah Derrett

Powered by Editorial Manager® and ProduXion Manager® from Aries Systems Corporation

<table>
<tr><td></td><td>Daniel Shepherd</td></tr>
<tr><td></td><td>David McBride</td></tr>
<tr><td>Opposed Reviewers:</td><td></td></tr>
<tr><td>Additional Information:</td><td></td></tr>
<tr><td>Question</td><td>Response</td></tr>
<tr><td>Financial Disclosure

Enter a financial disclosure statement that describes the sources of funding for the work included in this submission. Review the submission guidelines for detailed requirements. View published research articles from PLOS ONE for specific examples.

This statement is required for submission and will appear in the published article if the submission is accepted. Please make sure it is accurate.

Unfunded studies
Enter: The author(s) received no specific funding for this work.

Funded studies
Enter a statement with the following details:
• Initials of the authors who received each award
• Grant numbers awarded to each author
• The full name of each funder
• URL of each funder website
• Did the sponsors or funders play any role in the study design, data collection and analysis, decision to publish, or preparation of the manuscript?
• NO - Include this sentence at the end of your statement: The funders had no role in study design, data collection and analysis, decision to publish, or preparation of the manuscript.
• YES - Specify the role(s) played.

* typeset</td><td>Authors with funding:DI, AR, AS
Funders:Veterans Medical Research Trust Fund (No website)
Lottery Health
https://www.communitymatters.govt.nz/lottery-health-research/
The Royal New Zealand Returned and Services Association
www.rsa.org.nz
The funders had no role in study design, data collection and analysis, decision to publish, or preparation of the manuscript.</td></tr>
<tr><td>Competing Interests

Use the instructions below to enter a competing interest statement for this submission. On behalf of all authors, disclose any competing interests that could be perceived to bias this</td><td>The authors have declared that no competing interests exist.</td></tr>
</table>

work—acknowledging all financial support and any other relevant financial or non-financial competing interests.<br><br>This statement **will appear in the published article** if the submission is accepted. Please make sure it is accurate. View published research articles from *PLOS ONE* for specific examples.<br><br>**NO authors have competing interests**<br><br>Enter: *The authors have declared that no competing interests exist.*<br><br>**Authors with competing interests**<br><br>Enter competing interest details beginning with this statement:<br><br>*I have read the journal's policy and the authors of this manuscript have the following competing interests: [insert competing interests here]*<br><br>* typeset	
**Ethics Statement**<br><br>Enter an ethics statement for this submission. This statement is required if the study involved:<br><br>• Human participants<br>• Human specimens or tissue<br>• Vertebrate animals or cephalopods<br>• Vertebrate embryos or tissues<br>• Field research<br><br>Write "N/A" if the submission does not require an ethics statement.<br><br>General guidance is provided below. Consult the submission guidelines for detailed instructions. **Make sure that all information entered here is included in the Methods section of the manuscript.**	Southern Health and Disability Ethics Committee of New Zealand (15/STH/40/AM02).

Powered by Editorial Manager® and ProduXion Manager® from Aries Systems Corporation

**Format for specific study types**<br><br>**Human Subject Research (involving human participants and/or tissue)**<br>• Give the name of the institutional review board or ethics committee that approved the study<br>• Include the approval number and/or a statement indicating approval of this research<br>• Indicate the form of consent obtained (written/oral) or the reason that consent was not obtained (e.g. the data were analyzed anonymously)<br><br>**Animal Research (involving vertebrate animals, embryos or tissues)**<br>• Provide the name of the Institutional Animal Care and Use Committee (IACUC) or other relevant ethics board that reviewed the study protocol, and indicate whether they approved this research or granted a formal waiver of ethical approval<br>• Include an approval number if one was obtained<br>• If the study involved *non-human primates*, add *additional details* about animal welfare and steps taken to ameliorate suffering<br>• If anesthesia, euthanasia, or any kind of animal sacrifice is part of the study, include briefly which substances and/or methods were applied<br><br>**Field Research**<br><br>Include the following details if this study involves the collection of plant, animal, or other materials from a natural setting:<br>• Field permit number<br>• Name of the institution or relevant body that granted permission	
**Data Availability**<br><br>Authors are required to make all data underlying the findings described fully available, without restriction, and from the time of publication. PLOS allows rare exceptions to address legal and ethical concerns. See the PLOS Data Policy and FAQ for detailed information.	No - some restrictions will apply

A Data Availability Statement describing where the data can be found is required at submission. Your answers to this question constitute the Data Availability Statement and **will be published in the article**, if accepted.<br><br>**Important:** Stating 'data available on request from the author' is not sufficient. If your data are only available upon request, select 'No' for the first question and explain your exceptional situation in the text box.<br><br>Do the authors confirm that all data underlying the findings described in their manuscript are fully available without restriction?	
**Describe where the data may be found in full sentences. If you are copying our sample text, replace any instances of XXX with the appropriate details.**<br><br>• If the data are **held or will be held in a public repository**, include URLs, accession numbers or DOIs. If this information will only be available after acceptance, indicate this by ticking the box below. For example: *All XXX files are available from the XXX database (accession number(s) XXX, XXX.).*<br>• If the data are all contained **within the manuscript and/or Supporting Information files**, enter the following: *All relevant data are within the manuscript and its Supporting Information files.*<br>• If neither of these applies but you are able to provide **details of access elsewhere**, with or without limitations, please do so. For example:<br><br>*Data cannot be shared publicly because of [XXX]. Data are available from the XXX Institutional Data Access / Ethics Committee (contact via XXX) for researchers who meet the criteria for access to confidential data.*<br><br>*The data underlying the results presented in the study are available from (include the name of the third party*	All relevant data are within the manuscript and its Supporting Information files.

Powered by Editorial Manager® and ProduXion Manager® from Aries Systems Corporation

and contact information or URL).<br>• This text is appropriate if the data are owned by a third party and authors do not have permission to share the data.<br><br>* typeset	
Additional data availability information:	Tick here if the URLs/accession numbers/DOIs will be available only after acceptance of the manuscript for publication so that we can ensure their inclusion before publication.

Powered by Editorial Manager® and ProduXion Manager® from Aries Systems Corporation

UNIVERSITY of OTAGO

SAPERE – AUDE

Te Whare Wānanga o Otāgo

22 August 2019.

Preventive and Social Medicine,

University of Otago.

Dear Editors,

Kia ora,

We are submitting this article to PLoS One because it highlights risk and protective factors for post-traumatic stress, an important condition worldwide, and prevalent in our veteran community. Non-deployed veterans were included, so the risk factors include not only deployment related trauma, but trauma from other life events in the military setting. We have found that good sleep and resilience are both protective, which facts will help with prevention and treatment.

Some previously published work has looked at these conditions but have not taken such a holistic view.

Coming from New Zealand, the population in this cross sectional study is small, but our response rate compares favourably with other veteran studies: they can be difficult to engage with. Our strength lies in having veterans on the team. We also include findings from our Māori indigenous population, relatively well represented in the sample.

We have not previously submitted it to PLOS or any other journal.

The appropriate Academic Editors would be:

Erin Bouldin

Ann Marie Cheney

Briony Hill or;

Tracey Weiland

We have no opposing reviewers.

Ngā mihi,

David McBride

Dave McBride on behalf of the team

Tel 64 3 479 7208▪Fax 64 3 479 7298▪Mobile 64 27 253 5451

Email david.mcbride@otago.ac.nz

www.otago.ac.nz

MS Click here to download Manuscript Richardson PTS in NZ military.docx

Post-Traumatic Stress among New Zealand military personnel: a cross sectional study

Amy Richardson (BA, PGDipSci, PhD),[1] Gagan Gurung (PhD),[2] Ari Samaranayaka (BSc, MPhil, PhD),[3] Dianne Gardner (PhD),[4] Brandon deGraaf (BSc),[1] Emma H. Wyeth (BSc(Hons), PhD),[5] Sarah Derrett (BA, MPH, PhD),[1] Daniel Shepherd (BA, MSc, PhD)[6], David McBride (MB BCh BAO, PhD)[1*]

[1]Injury Prevention Research Unit, Department of Preventive and Social Medicine, Dunedin School of Medicine, University of Otago, New Zealand

[2]Department of Preventive and Social Medicine, Dunedin School of Medicine, University of Otago, New Zealand

[3]Centre for Biostatistics, Division of Health Sciences, University of Otago, New Zealand

[4]School of Psychology, Massey University, New Zealand

[5]Ngāi Tahu Māori Health Research Unit, Department of Preventive and Social Medicine, Dunedin School of Medicine, University of Otago, New Zealand

[6] Department of Psychology, Auckland University of Technology, New Zealand

Corresponding Author:

Email: david.mcbride@otago.ac.nz

Phone: 64 3 479 7208

Abstract

Background: Post-traumatic stress (PTS) is prevalent among military personnel. Knowledge of the protective and harmful factors associated with PTS in this population may assist with identifying personnel who would benefit from increased or targeted support.

Aims: To examine factors associated with PTS among New Zealand military personnel.

Methods: For this cross-sectional study, currently serving and retired military personnel were invited to complete a questionnaire. The questionnaire included a measure of PTS (the Military Post-traumatic Stress Disorder Checklist; PCL-M), where scores ≥30 indicate the experience of significant PTS symptoms and scores ≥45 indicate a presumptive clinical diagnosis of post-traumatic stress. Potentially protective and harmful factors associated with PTS were examined using logistic regression modelling.

Results: 1817 military personnel completed the questionnaire. PCL-M scores were ≥30 for 549 (30%) participants and ≥45 for 179 (10%) participants. Exposure to trauma was most strongly associated with PCL-M scores ≥45 (OR 3.34, 95% CI 1.54-7.27, p<0.01). Higher PCL-M scores were also associated with older age, male sex, and Māori ethnicity (New Zealand's indigenous population). Factors associated with lower PCL-M scores were greater length of service, psychological flexibility, and better quality sleep.

Conclusions: PTS was found to be prevalent among New Zealand military personnel. The experience of trauma was strongly associated with PTS. However, factors such as psychological flexibility (the ability to adapt to changes in circumstances) and good sleep were protective, suggesting that these factors could be key targets for interventions designed to reduce PTS among military personnel in New Zealand.

Key words

Military Personnel, Post-Traumatic Stress (PTS), Psychological Flexibility, Sleep, Trauma

Introduction

In New Zealand, the Defence Force has three primary personnel groups: the Regular Force, Reserve Forces, and Civilians (including those employed by the Defence Force and working overseas) (1). These military personnel are responsible for contributing to the defence, security and wellbeing of the country. Research from other countries suggests that while many military personnel cope well with their roles (2) , they are exposed to higher rates of both military and non-military trauma compared to the general population (3-5) , and are at greater risk of experiencing post-traumatic stress (PTS) (6). An elevated risk has been identified among military personnel even during periods of low deployment activity (7-9).

Two critical events are commonly described in the military 'life course' – achievement of veteran status through operational deployment, and transition from military to civilian life. Operational deployment and witnessing atrocities has been associated with PTS (10, 11), while the period of transition from the military to civilian life has been found to confer an elevated risk of suicide, regardless of deployment history (12). A lack of support during this period (including social and family support) serves to amplify suicide risk, and has been found to contribute to the experience of PTS (13).

While the critical events described above have been identified across diverse military samples (13), estimates of the prevalence of PTS vary dramatically both across and within countries. For example, the prevalence of PTS identified within the United Kingdom Armed Forces is significantly lower than that reported for military personnel serving in the United States, Australia, and Canada (14). Although differences are partially attributable to variation in sampling strategies, research methods, and diagnostic thresholds, differences in exposure to risk factors are also likely to play a role (14). Research in veteran populations suggests that factors that can negatively affect adaptation to deployment and civilian transition include: female gender, ethnicity, high number of and longer duration deployments, prior adverse life events, pre-existing psychological disorders, trauma exposure, and alcohol misuse (13, 15-18). Conversely, sleep (19, 20), social support (21), and psychological flexibility (the ability to

fully experience thoughts and feelings and flexibly choose behaviour that is in line with personal goals and values) (22) have potential to protect against poor mental health outcomes, including PTS.

A significant number of New Zealand military personnel have been exposed to high levels of combat-related trauma and others have been deployed on peace-keeping missions (23). Moral injury is one stressor associated with peace-keeping missions, defined as "perpetrating, failing to prevent, bearing witness to, or learning about acts that transgress deeply held moral beliefs and expectations" (24). Other stressors include the restrictive rules of engagement (25), monotony and boredom, personnel encounter difficulties, and separation from family (26). Pre-deployment and follow-up stages are also important to consider; these were the most stressful periods, and had the greatest effect on mental and physical health, in a longitudinal study of 277 New Zealand military personnel deployed on peacekeeping duties (23).

While 10% of a community sample of New Zealand Vietnam War veterans were found to experience PTS (27), the prevalence of PTS among New Zealand military personnel more generally has not previously been reported. One reason for this is lower response rates to post-deployment screens for PTS (28). Research is also yet to identify the key harmful and protective factors associated with PTS in this population. This information is important to detect individuals who would benefit from targeted support following transition from the military, in order to reduce their risk of experiencing PTS. The aims of this cross-sectional study were to: 1) determine the prevalence of PTS (symptomology and presumptive cases), and 2) identify protective and harmful factors most strongly associated with PTS in New Zealand military personnel.

Materials and Methods

Sample

An online or paper questionnaire was available during June to December 2018 for completion by all currently serving and retired military personnel living in New Zealand. We attempted to minimise bias through an intensive recruitment campaign. In mid-June, a link to the online questionnaire was sent by email to all currently serving regular and reserve New Zealand Defence Force (NZDF) members who

were holders of the New Zealand Operational Service Medal (NZOSM), numbering 3874 individuals at that time. A global message and a link to the questionnaire was also presented on the NZDF 'intranet landing page', the webpage from which all currently serving personnel can access relevant work-related content, tools, and resources. Retired military personnel were recruited through paper questionnaires and posters distributed to the 43 local social clubs of the Royal New Zealand Returned and Services Association (RSA) identified by the RSA national office to be 'veteran active.' Announcements were also made on military social media pages, and both retired and currently serving personnel were invited to participate through an announcement on the No Duff Charitable Trust website; No Duff is a registered charity committed to providing confidential support for military personnel and their families in New Zealand (29).

Procedure

Military personnel interested in taking part in the study were directed to visit a website where they were required to enter their name and email address. This resulted in a personalised link being sent to their email address from which they could complete an online secure version of the questionnaire. All serving military personnel were informed that they could request a paper version of the survey if this was their personal preference. The study received approval from the Southern Health and Disability Ethics Committee of New Zealand (15/STH/40/AM02). We consulted with the Ngāi Tahu Research Consultation Committee in order to assess the importance of the project to Māori, New Zealand's indigenous population. For 6 predictor variables in a multivariate model, we needed at least 600 cases.

Measures

The questionnaire included standardised measures of PTS (outcome), and potentially harmful and protective exposures. Potentially adverse exposures examined included trauma, general distress, and hazardous drinking. Protective exposures included social support, sleep, and psychological flexibility.

Symptoms of PTS were assessed using the post-traumatic stress disorder (PTSD) checklist – military version (PCL-M). The PCL-M includes 17 items that ask about symptoms related to stressful military

experiences, with response options ranging from 1 ‘Not at all’ to 5 ‘Extremely’ (30). A total symptom severity score is calculated by summing responses to each option. While scores of 30-35 indicate significant PTS symptomology and probable cases of PTSD, scores of ≥45 indicate a presumptive PTSD diagnosis (30). The PCL-M has been identified as a widely used and well-validated measure (31).

The Brief Trauma Questionnaire (BTQ) was used to assess exposure to trauma. The BTQ consists of 10 items that screen for a range of different traumatic experiences [31]. Exposure to an event is scored as positive if a respondent says ‘yes’ to indicate life threat or serious injury from combat trauma, a serious car accident, a natural disaster, life-threatening illness, and physical or sexual abuse, or to indicate exposure to violent death (32). The BTQ is considered a reliable and valid measure to assess trauma exposure in defence force personnel (33).

Symptoms of distress were screened for using the General Health Questionnaire 12 (GHQ-12). This measure includes 12 items with a four-point response scale (34). Items are summed to yield an overall total score, with higher scores indicating greater distress (34).

The AUDIT-C is a 3-item measure that was used to identify potentially hazardous drinking (35). Each item is answered using five response options, with possible total scores ranging from 0 to 12 (35). A total score of ≥3 for women, and ≥4 for men, was used to identify participants engaging in hazardous drinking. The AUDIT-C has been validated in veteran populations (36, 37).

The Social Provisions Scale (SPS) was used to examine participant perceptions of the availability of different dimensions of social support. This theory-based social support instrument includes 24 items distributed across six subscales: reliable alliance, attachment, nurturance, social integration, reassurance of worth, and guidance (38). Each item is rated on a 4-point Likert scale with responses ranging from ‘strongly disagree’ to ‘strongly agree’ (38). After reversal of negatively worded items a total score was computed by summing all items. Higher scores (including total scores and individual subscale scores) indicate higher levels of perceived social support. The construct validity, internal

consistency, and test-retest reliability of the measure has been established across diverse populations (38, 39).

To screen for insomnia disorder based on DSM-5 criteria we used the 8-item Sleep Condition Indicator (SCI) (40). All items were scored on a four point scale (0 to 4), with possible summed scores on this measure range from 0 to 32; scores were then re-scaled to a 0 to 10 scale (40). Higher scores are indicative of better sleep.

To evaluate psychological flexibility, the 10-item Acceptance and Action Questionnaire II (AAQ-II) was used (41). After reversing negatively worded items, the items of the scale were summed to obtain a total score (possible range 10 to 70), with higher scores indicative of greater psychological flexibility (less experiential avoidance) (41).

The questionnaire also included a series of sociodemographic questions (gender, ethnicity, marital status, education level, employment status) and questions about service history, rank, and deployments.

Analyses

Statistical analyses were completed using Stata 15 software (42). First, descriptive statistics were used to describe the demographic characteristics of participants. Next, exploratory analyses investigated univariate associations between demographic, hazardous and protective factors, and the PTS outcomes (PCL-M ≥30 and PCL-M ≥45), with odds ratios (ORs) and 95% confidence intervals (CIs) estimated using logistic regression. Following this, multivariable logistic regression (adjusted for age, sex, service years, and deployment status) was performed to identify exposures associated with PTS using a backward elimination process; variables with a *p*-value >0.10 were sequentially dropped from the model. Missing data was addressed using developer recommendations or previous approaches for each measure. For example, in instances where one item was missing from the PCL-M the mean score was imputed; if more than one item was missing on the PCL-M the case was excluded from analyses (43). Only participants with complete data were included in the multivariable analyses.

Results

A total of 1817 military personnel completed the questionnaire; 90 of the participants completed a paper version. Among participants, 549 (30%) reported PCL-M scores of ≥30 indicating significant PTS symptomology (probable PTSD), and 179 (10%) reported PCL-M scores of ≥45, indicative of presumptive clinical PTS.

The demographic characteristics of participants categorised according to the experience of probable PTS (scores ≥30) are presented in Table 1.

Table 1. Demographic characteristics of participants according to PCL-M scores.

Characteristic	Low PCL-M Score 17-29 (*n* = 1268)	High PCL-M Score ≥30 (*n* = 549)
Age (years)		
20-29	124 (84%)	24 (16%)
30-39	264 (75%)	86 (25%)
40-49	327 (71%)	134 (29%)
50-59	247 (69%)	111 (31%)
60-69	176 (63%)	103 (37%)
70+	127 (59%)	89 (41%)
missing	3 (60%)	2 (40%)
Sex		
Female	183 (77%)	54 (23%)
Male	1065 (69%)	488 (31%)
missing	20 (74%)	7 (26%)
Ethnicity		
NZ European	997 (70%)	418 (30%)
Māori	177 (69%)	79 (31%)
Other	94 (64%)	52 (36%)
Service Years		
0-9	213 (62%)	132 (38%)
10-19	350 (70%)	153 (30%)
20-29	390 (73%)	144 (27%)
30-39	181 (69%)	80 (31%)
40-49	43 (65%)	23 (35%)
missing	91 (84%)	17 (16%)
Deployed		
No	186 (67%)	92 (33%)
Yes	1012 (71%)	415 (29%)
missing	70 (63%)	42 (37%)

The median age of participants was 49.1 years (interquartile range = 38.7 – 61.1 years). The majority were male (87%) and were of New Zealand European ethnicity (78%); 14% of participants identified as Māori, similar to that of the NZDF as a whole, reported as 15%. Most participants had served in the military for at least 10 years (80%) and had been deployed at least once (84%).

Descriptive statistics for exposures (harmful and protective factors) treated as continuous variables in the analyses are presented in Table 2.

Table 2. Descriptive statistics for continuous variables used in logistic regression analyses.

**Exposure**	**Mean**	**Standard Deviation**	**Median**	**Inter-quartile Range**
Distress (GHQ-12)<br>*n* = 1735	11.9	5.1	11	8 – 14
Social Support (SPS)<br>*n* = 1778	75.8	10.8	75	40 – 96
Psychological Flexibility (AAQ-II)<br>*n* = 1734	52.3	10.1	54	46 – 60
Sleep (SCI)<br>*n* = 1711	5.9	2.2	5.6	4.4 – 7.8

Of the 1656 participants who completed the AUDIT-C, 898 (54%) reported hazardous drinking. Of the 1715 participants who completed the BTQ, the majority (*n* = 1187, 69%) had been exposed to trauma. 1006 (59%) had served in a war zone and 736 (73%) of these individuals reported that this presented a threat to life and/or a threat of serious injury. The proportion of participants experiencing other traumatic events, including childhood physical and sexual abuse, was also high (35% and 16% respectively).

Univariate Analyses

Results of univariate analyses describing associations between exposure variables (demographic, risk, and protective factors) and PTS are presented in Table 3, showing both the odds of experiencing symptoms of PTS (PCL-M scores ≥30) and the odds of experiencing clinically relevant PTS (scores ≥45). With respect to PTS symptomology, older age, male sex, higher distress, and exposure to trauma were significantly associated with increased likelihood of PTS symptoms. In contrast, increased number of years in service, social support, psychological flexibility, and sleep were significantly associated with lower odds of experiencing PTS symptoms. The same pattern of associations was also found for clinically relevant PTS, in addition to greater odds of clinical PTS among individuals identifying as Māori compared to those of NZ European ethnicity.

Table 3. Univariate associations between exposure variables and elevated PCL-M scores (≥30 and ≥45 respectively).

**Characteristic**	**PCL-M Score ≥30**				**PCL-M Score ≥45**			
	**OR**	**95% CI for OR**	***p***	***n***	**OR**	**95% CI for OR**	***p***	***n***
*Age (Years)**	1.02	1.01, 1.03	<0.01	1812	1.03	1.02, 1.05	<0.01	1812
Sex								
Female	Ref				Ref			
Male	1.55	1.13, 2.14	0.01	1790	2.03	1.14, 3.64	0.02	1790
Ethnicity								
NZ European	Ref				Ref			
Māori	1.06	0.80, 1.42	0.67		1.78	1.21, 2.63	<0.01	
Other	1.32	0.92, 1.89	0.13	1817	1.17	0.67, 2.06	0.58	1817
*Service Years**	0.99	0.98, 1.00	0.03	1709	0.97	0.96, 0.99	<0.01	1709
Deployed								
No	Ref				Ref			
Yes	0.83	0.63, 1.10	0.18	1705	0.76	0.50, 1.13	0.18	1705
*Distress**	1.27	1.23, 1.30	<0.01	1735	1.21	1.18, 1.25	<0.01	1735
*Social Support**	0.91	0.90, 0.93	<0.01	1778	0.91	0.90, 0.93	<0.01	1778
*Psychological Flexibility**	0.84	0.82, 0.85	<0.01	1734	0.84	0.82, 0.86	<0.01	1734
*Sleep**	0.53	0.49, 0.57	<0.01	1711	0.41	0.36, 0.46	<0.01	1711
Hazardous Drinking								
No	Ref				Ref			
Yes	1.06	0.86, 1.31	0.58	1656	0.81	0.58, 1.12	0.20	1656
Trauma Exposure								
No	Ref				Ref			
Yes	4.04	3.05, 5.35	<0.01	1715	7.11	3.82, 13.23	<0.01	1715

Note. *Continuous variable (no reference group).

Multivariate Analyses

Results of multivariate analyses describing associations between exposure variables and PTS, after adjustment for age, sex, service years and deployment status, are presented in Table 4, including for odds of experiencing PTS symptomology (PCL-M scores ≥30) and for odds of clinically relevant PTS (scores ≥45). With respect to PTS symptomology, older age, male sex, higher distress, and exposure to trauma were significantly associated with an increased likelihood of PTS. Increased number of years in service, psychological flexibility, and sleep were significantly associated with decreased odds of experiencing PTS symptoms; social support was no longer significantly associated with this outcome. A single unit increase in sleep score corresponded to a 30% reduction in odds of experiencing significant PTS symptoms. The same pattern of results was found for clinically-relevant PTS, with the exception of higher distress, which was not significantly associated. In addition, Māori participants were found to have greater odds of experiencing clinically relevant PTS when compared to New Zealand Europeans.

Table 4. Multivariate associations between exposure variables and elevated PTSD (scores ≥ 30 and scores ≥ 45).

**Characteristic**	**PCL-M Score ≥30, n = 1532**			**PCL-M Score ≥45, n = 1567**		
	**OR**	**95% CI for OR**	***p***	**OR**	**95% CI for OR**	***p***
*Age (Years)**	1.02	1.01, 1.03	<0.01	1.04	1.03, 1.06	<0.01
Sex						
Female	Ref			Ref		
Male	1.84	1.14, 2.98	0.01	1.69	0.74, 3.86	0.21
Ethnicity						
NZ European				Ref		
Māori				2.80	1.54, 5.10	<0.01
Other				0.97	0.40, 2.31	0.94
*Service Years**	0.98	0.97, 1.00	0.01	0.97	0.95, 0.99	<0.01
Deployed						
No	Ref			Ref		
Yes	1.31	0.85, 2.00	0.22	1.54	0.84, 2.81	0.16
*Distress**	1.07	1.03, 1.11	<0.01			
*Psychological Flexibility**	0.87	0.85, 0.89	<0.01	0.87	0.85, 0.90	<0.01
*Sleep**	0.70	0.64, 0.77	<0.01	0.56	0.49, 0.66	<0.01
Hazardous Drinking						
No	Ref					
Yes	1.11	0.96, 1.77	0.08			
Trauma Exposure						
No	Ref			Ref		
Yes	3.03	2.07, 4.41	<0.01	3.34	1.54, 7.27	<0.01

Note. Variables with a *p*-value less than 0.10 after adjustment for age, sex, service years, and deployment status are included in the model. *Continuous variable (no reference group).

Discussion

This cross-sectional study identified a high prevalence of PTS in a large sample of currently serving and retired New Zealand military personnel. Thirty percent of participants reported experiencing probable PTS and 10% were identified as having clinically relevant PTS. These findings are similar to those reported in an earlier study, which found evidence of PTS among 10% of New Zealand Vietnam war veterans (27), indicating that it is not only retired veterans who are at risk. Our results highlight that support to deal with PTS is needed for a large number of New Zealanders who are serving, or have served, in the military.

Strengths of this study include the large sample, and the inclusion of those who have never been deployed. Our study also serves to provide a snapshot of New Zealand military personnel, for which the total number of those who have served and who are currently serving is unknown. Although the number of individuals in the sample who had never been deployed was small, our findings suggest that these individuals are also at risk of PTS. Evidence that personnel who have never deployed are at greater risk of PTS than the general population is growing (9, 44). It is clear that factors other than deployment have an important role to play in the experience of PTS among military personnel.

At the time the questionnaire was distributed there were 4822 serving veterans in the NZDF, 1038 of whom were reservists and unlikely to receive the email invitation. As the majority of questionnaires were completed by currently serving personnel, the response rate would have been in the order of 30%. This gives rise to potential sampling bias. Military personnel with higher PTS may have been more likely to participate, giving rise to inflated estimates of PTS prevalence. Conversely, our findings may underestimate the prevalence of PTS in this population if those without PTS were more inclined to participate. Another limitation of the study is the cross-sectional design which precludes the identification of cause and effect relationships between exposures and PTS. Additionally, although we assessed and accounted for a range of known confounders, it is still possible some important

confounders were not assessed in the survey and may explain significant relationships between exposures and PTS.

It is unclear how generalisable the study findings are to countries outside of New Zealand, where the characteristics of military personnel, deployment experiences, and post-deployment support services are likely to differ (14). Nevertheless, health support for military personnel in New Zealand follows that provided by American, Canadian, British, and Australian Defence Forces. Scores on the PCL-M measure reported by our participants suggest that as many as 10% may have symptoms likely to result in a clinical diagnosis of PTSD. This is in line with point prevalence estimates of combat-related PTSD in US military veterans, ranging from approximately 2% to 17% (6). Summary estimates of PTSD prevalence for military personnel and veterans from a number of countries range from 1.1% to 34.8% (13). The prevalence of PTS identified in our study is higher than that documented among UK military personnel, although this may be attributable to variation in sampling strategies and measures used (45). Differences in PTS estimates may also be due to variation in socio-political and cultural factors that vary across nations (6).

Trauma exposure was most strongly associated with odds of experiencing PTS symptomology and clinically relevant PTS in the present study, and is a prerequisite for a DSM IV diagnosis of PTSD. General distress was significantly associated with increased odds of PTS symptoms, although was not significantly associated with odds of clinical PTS after adjustment for age, sex, service years, and deployment status. This is consistent with findings from a meta-analysis of risk factors for combat-related PTS among military personnel and veterans, which did not identify general distress to be a significant risk factor, although a history of prior psychological problems and trauma exposure were (13).

Māori participants had greater odds of reporting clinical PTS than their New Zealand European counterparts. Higher levels of PTS among Māori were also detected in a sample of 756 New Zealand Vietnam War veterans, however, the effect of ethnicity on PTS was mediated by higher levels of

combat stressors experienced by Māori, including stressors related to combat exposure, rank, and combat role (46).

Consistent with findings of a study examining predictors of persistent PTS in UK military personnel (47), older age was significantly associated with increased odds of experiencing PTS. However, in contrast to other studies examining PTS in military personnel, males were at greater risk of experiencing PTS than females. It is important to note that other studies identifying females to be at greater risk of PTS have focused on combat-related PTS (13), and our sample includes personnel who never deployed.

Despite older age being associated with increased odds of PTS, a greater number of service years was associated with reduced odds of PTS. This may reflect a resilience that develops over time among long-serving personnel, or may be due to individuals with PTS leaving military service earlier as has been reported among UK Armed Forces Personnel (48). Sleep was also found to be protective against PTS. Self-reported sleep problems have consistently been associated with the experience of PTS in veteran populations (49). Early intervention among military personnel experiencing sleep disturbance may help to reduce PTS symptoms. An investigation of 44 military personnel who received cognitive behavioural therapy for insomnia found that participants who experienced improved sleep quality from pre- to post-treatment (n=28) had significant declines in depression and PTS symptoms (20). In contrast, those whose sleep did not improve had no changes in their psychiatric symptoms, as well as a reduction in health-related quality of life.

In the present study, psychological flexibility was associated with reduced odds of reporting PTS. However, it is important to interpret our findings with caution as there have been criticisms of the AAQ-II, with several researchers arguing that it may be measuring psychological distress rather than psychological inflexibility (50). Furthermore, numerous versions of the AAQ-II exist which can make it difficult to compare findings across studies. Despite few studies examining psychological flexibility among military personnel, techniques designed to increase psychological flexibility (such as

acceptance and commitment therapy) are being investigated as potential treatments for PTS in this population (51-53), and evidence for their effectiveness is emerging (54).

Conclusions

Knowledge of the harmful and protective factors associated with PTS may allow for early identification of military personnel who would benefit from targeted support to promote their wellbeing. Our findings suggest that traumatic exposure is most strongly associated with high levels of PTS, while good sleep and psychological flexibility are protective. As these protective factors are amenable to standardised measurement and modification, early detection could facilitate intervention. Future research is needed to identify whether relationships can be found longitudinally, which would provide further evidence regarding New Zealand military personnel most at risk of experiencing PTS.

Acknowledgements

The authors would like to thank all New Zealand military personnel who participated in this study.

The study was funded by the Veterans Medical Research Trust Fund, Lottery Health and the Royal New Zealand Returned and Services Association.

References

1. New Zealand Defence Force. Personnel Summary: New Zealand Government; 2019. Available from: http://www.nzdf.mil.nz/personnel-records/personnel-branch/.

2. Hunt EJF, Wessely S, Jones N, Rona RJ, Greenberg N. The mental health of the UK Armed Forces: where facts meet fiction. Eur J Psychotraumatol. 2014;5(1): 23617.

3. Stretch RH, Knudson KH, Durand D. Effects of premilitary and military trauma on the development of post-traumatic stress disorder symptoms in female and male active duty soldiers. Mil Med. 1998;163(7): 466-470.

4. Durand D, Knudson KH, Stretch RH. Psychological Health and Trauma in Male and Female Soldiers. Mil Med. 1998;163(6): 363-367.

5. Afifi TO, Taillieu T, Zamorski MA, Turner S, Cheung K, Sareen J. Association of child abuse exposure with suicidal ideation, suicide plans, and suicide attempts in military personnel and the general population in Canada. JAMA Psychiatry. 2016;73(3): 229-238.

6. Richardson LK, Frueh BC, Acierno R. Prevalence estimates of combat-related post-traumatic stress disorder: critical review. Aust N Z J Psychiatry. 2010;44(1): 4-19.

7. Roux Cl, Stein DJ, Seedat S. Prevalence and characteristics of trauma and post-traumatic stress symptoms in operational members of the South African National Defence Force. Mil Med. 2003;168(1): 71-75.

8. Jones M, Rona RJ, Hooper R, Wesseley S. The burden of psychological symptoms in UK Armed Forces. Occup Med. 2006;56(5): 322-328.

9. McKenzie DP, Ikin JF, McFarlane AC, Creamer M, Forbes AB, Kelsall HL, et al. Psychological health of Australian veterans of the 1991 Gulf War: an assessment using the SF-12, GHQ-12 and PCL-S. Psychol Med. 2004;34(8): 1419-1430.

10. Jones M, Sundin J, Goodwin L, Hull L, Fear NT, Wessely S, et al. What explains post-traumatic stress disorder (PTSD) in UK service personnel: deployment or something else? Psychol Med. 2012;43(8): 1703-1712.

11. Sareen J, Cox BJ, Afifi TO, Stein MB, Belik S-L, Meadows G, et al. Combat and peacekeeping operations in relation to prevalence of mental disorders and perceived need for mental health care: findings from a large representative sample of military personnel. JAMA Psychiatry. 2007;64(7): 843-852.

12. Pease JL, Billera M, Gerard G. Military culture and the transition to civilian life: suicide risk and other considerations. Social Work. 2015;61(1): 83-86.

13. Xue C, Ge Y, Tang B, Liu Y, Kang P, Wang M, et al. A Meta-Analysis of Risk Factors for Combat-Related PTSD among Military Personnel and Veterans. PLoS One. 2015;10(3): e0120270.

14. Yehuda R, Vermetten E, McFarlane AC, Lehrner A. PTSD in the military: special considerations for understanding prevalence, pathophysiology and treatment following deployment. Eur J Psychotraumatol. 2014;5(1): 25322.

15. Koenen KC, Stellman SD, Dohrenwend BP, Sommer Jr. JF, Stellman JM. The consistency of combat exposure reporting and course of PTSD in Vietnam War veterans. J Trauma Stress. 2007;20(1): 3-13.

16. Reger MA, Gahm GA, Swanson RD, Duma SJ. Association between number of deployments to Iraq and mental health screening outcomes in US Army soldiers. J Clin Psychiatry. 2009;70(9): 1266-1272.

17. Jakupcak M, Tull MT, McDermott MJ, Kaysen D, Hunt S, Simpson T. PTSD symptom clusters in relationship to alcohol misuse among Iraq and Afghanistan war veterans seeking post-deployment VA health care. Addict Behav. 2010;35(9): 840-843.

18. Iversen A, Waterdrinker A, Fear N, Greenberg N, Barker C, Hotopf M, et al. Factors associated with heavy alcohol consumption in the U.K. Armed Forces: data from a health survey of Gulf, Bosnia, and era Veterans. Mil Med. 2007;172(9): 956-961.

19. Wright KM, Britt TW, Bliese PD, Adler AB, Picchioni D, Moore D. Insomnia as predictor versus outcome of PTSD and depression among Iraq combat veterans. J Clin Psychol. 2011;67(12): 1240-1258.

20. Rusch HL, Guardado P, Baxter T, Mysliwiec V, Gill JM. Improved sleep quality is associated with reductions in depression and PTSD arousal symptoms and increases in IGF-1 concentrations. J Clin Sleep Med. 2015;11(6): 615-623.

21. Pietrzak RH, Johnson DC, Goldstein MB, Malley JC, Southwick SM. Psychological resilience and postdeployment social support protect against traumatic stress and depressive symptoms in soldiers returning from Operations Enduring Freedom and Iraqi Freedom. Depress Anxiety. 2009;26(8): 745-751.

22. Dutra SJ, Sadeh N. Psychological flexibility mitigates effects of PTSD symptoms and negative urgency on aggressive behavior in trauma-exposed veterans. Personality Disord. 2018;9(4): 315-23.

23. Chamberlain K, MacDonald C, Pereira-Laird J, Long N, Mirfin K. Mental health, physical health, and stressors reported by New Zealand Defence Force peacekeepers: a longitudinal study. Mil Med. 1998;163(7): 477-481.

24. Litz BT, Stein N, Delaney E, Lebowitz L, Nash WP, Silva C, et al. Moral injury and moral repair in war veterans: A preliminary model and intervention strategy. Clin Psychol Rev. 2009;29(8): 695-706.

25. Litz BT, Orsillo SM, Friedman M, Ehlich P, et al. Posttraumatic stress disorder associated with peacekeeping duty in Somalia for U.S. military personnel. Am J Psychiatry. 1997;154(2): 178-184.

26. Ritchie EC, Ruck DC, Anderson MW. The 528th combat stress control unit in Somalia in support of operation restore hope. Mil Med. 1994;159(5): 372-376.

27. Long N, MacDonald C, Chamberlain K. Prevalence of posttraumatic stress disorder, depression and anxiety in a community sample of New Zealand Vietnam War veterans. Aust N Z J Psychiatry. 1996;30(2): 253-256.

28. Brounéus K, Wray M, Green P. Underestimating the burden for peacekeepers? Difficulty in determining psychological well-being following operational deployment with low response rates from NZDF personnel. J Mil Veterans Health. 2015;23(2): 7-13.

29. No Duff Charitable Trust. No Duff - Confidential help for veterans their families. 2019. Available from: https://www.noduff.org/.

30. U.S. Department of Veterans Affairs. Using the PTSD Checklist (PCL). Veterans Affairs National Center for PTSD; 2012.

31. Wilkins KC, Lang AJ, Norman SB. Synthesis of the psychometric properties of the PTSD checklist (PCL) military, civilian, and specific versions. Depress Anxiety. 2011;28(7): 596-606.

32. Schnurr PP, Spiro A, Vielhauer MJ, Findler MN, Hamblen JL. Trauma in the lives of older men: findings from the normative aging study. J Clin Geropsychol. 2002;8(3): 175-187.

33. Whealin JM, Batzer WB, Morgan CA, III, Detwiler HF, Jr., Schnurr PP, Friedman MJ. Cohesion, burnout, and past trauma in tri-service medical and support personnel. Mil Med. 2007;172(3): 266-272.

34. Goldberg D, Williams P. A user's guide to the General Health Questionnaire. Windsor, United Kingdom: NFER-Nelson; 1988.

35. Bush K, Kivlahan DR, McDonell MB, Fihn SD, Bradley KA. The AUDIT alcohol consumption questions (AUDIT-C): an effective brief screening test for problem drinking. JAMA Intern Med. 1998;158(16): 1789-1795.

36. Crawford EF, Fulton JJ, Swinkels CM, Beckham JC, Calhoun PS. Diagnostic efficiency of the AUDIT-C in U.S. veterans with military service since September 11, 2001. Drug Alcohol Depend. 2013;132(1): 101-106.

37. Bradley KA, Bush KR, Epler AJ, Dobie DJ, Davis TM, Sporleder JL, et al. Two brief alcohol-screening tests from the Alcohol Use Disorders Identification Test (AUDIT): validation in a female Veterans Affairs patient population. JAMA Intern Med. 2003;163(7): 821-829.

38. Cutrona CE, Russell DW. The provisions of social relationships and adaptation to stress. Adv Pers Rel. 1987;1: 37-67.

39. Mancini JA, Blieszner R. Social provisions in adulthood: concept and measurement in close relationships. J Gerontol. 1992;47(1): P14-P20.

40. Espie CA, Kyle SD, Hames P, Gardani M, Fleming L, Cape J. The Sleep Condition Indicator: a clinical screening tool to evaluate insomnia disorder. BMJ Open. 2014;4(3): e004183.

41. Bond FW, Hayes SC, Baer RA, Carpenter KM, Guenole N, Orcutt HK, et al. Preliminary psychometric properties of the Acceptance and Action Questionnaire–II: a revised measure of psychological inflexibility and experiential avoidance. Behav Therapy. 2011;42(4): 676-688.

42. StataCorp. Stata Statistical Software: Release 15. College Station, TX: StataCorp LLC; 2017.

43. Iwasa H, Suzuki Y, Shiga T, Maeda M, Yabe H, Yasumura S. Psychometric evaluation of the Japanese version of the Posttraumatic Stress Disorder Checklist in community dwellers following the Fukushima Daiichi nuclear power plant incident: the Fukushima health management survey. SAGE Open. 2016;6(2): 2158244016652444.

44. Hoge CW, Castro CA, Messer SC, McGurk D, Cotting DI, Koffman RL. Combat duty in Iraq and Afghanistan, mental health problems, and barriers to care. New Eng J Med. 2004;351(1): 13-22.

45. Iversen AC, van Staden L, Hughes JH, Browne T, Hull L, Hall J, et al. The prevalence of common mental disorders and PTSD in the UK military: using data from a clinical interview-based study. BMC Psychiatry. 2009;9(1): 68.

46. MacDonald C, Chamberlain K, Long N. Race, combat, and PTSD in a community sample of New Zealand Vietnam war veterans. J Traum Stress. 1997;10(1): 117-124.

47. Rona RJ, Jones M, Sundin J, Goodwin L, Hull L, Wessely S, et al. Predicting persistent posttraumatic stress disorder (PTSD) in UK military personnel who served in Iraq: a longitudinal study. J Psychiat Res. 2012;46(9): 1191-1198.

48. Buckman JEJ, Forbes HJ, Clayton T, Jones M, Jones N, Greenberg N, et al. Early Service leavers: a study of the factors associated with premature separation from the UK Armed Forces and the mental health of those that leave early. Eur J Public Health. 2012;23(3): 410-415.

49. Baird T, McLeay S, Harvey W, Theal R, Law D, O'Sullivan R, et al. Sleep disturbances in Australian Vietnam veterans with and without posttraumatic stress disorder. J Clin Sleep Med. 2018;14(5): 745-752.

50. Wolgast M. What does the Acceptance and Action Questionnaire (AAQ-II) really measure? Behav Ther. 2014;45(6): 831-839.

51. Vujanovic AA, Niles B, Pietrefesa A, Schmertz SK, Potter CM. Mindfulness in the treatment of posttraumatic stress disorder among military veterans. Spiritual Clin Pract. 2013;1(S): 15-25.

52. King AP, Erickson TM, Giardino ND, Favorite T, Rauch SAM, Robinson E, et al. A pilot study of group mindfulness-based cognitive therapy (MBCT) for combat veterans with posttraumatic stress disorder (PTSD). Depress Anxiety. 2013;30(7): 638-645.

53. Casselman RB, Pemberton JR. ACT-based parenting group for veterans with PTSD: development and preliminary outcomes. Am J Fam Ther. 2015;43(1): 57-66.

54. Boyd JE, Lanius RA, McKinnon MC. Mindfulness-based treatments for posttraumatic stress disorder: a review of the treatment literature and neurobiological evidence. J Psychiatry Neurosci. 2018; 43(1): 7-25.

Strobe checklist

Click here to access/download

Supporting Information

Strobe checklist Richardson PTS in NZ.docx
